# Editorial on the special issue of Chinese Medicine for the 4th Sino-CPLP Symposium on Natural Medicine and Biodiversity Resources (SNMBR) & The International Forum on Research and Development of Traditional Chinese Medicine Industry (Macau) on Medicinal Plant Resource, Natural Product and Function, Pharmacology and Regulatory

**DOI:** 10.1186/s13020-023-00828-3

**Published:** 2023-11-02

This special issue contains 14 abstracts selected from the submitted conference papers in the 4th Sino-CPLP Symposium on Natural Medicine and Biodiversity Resources (SNMBR) & The International Forum on Research and Development of Traditional Chinese Medicine Industry (Macau), held at the University of Macau, Macau (SAR of China) on December 2–5, 2022.

Abstracts were chosen from more than 150 studies that were either presented orally or through posters at the conference, and were evaluated by a standard single-blind peer-review system consisting of two or more experts to evaluate the scientific coherence and clarity of the submitted manuscripts operated by *Chinese Medicine*. The Editorial Board further validated the manuscripts for publication.

This thematic collection contains various aspects of fundamental investigation of Chinese medicine, such as studying the anti-thrombosis effects of active compounds from traditional Chinese medicine (TCM) (e.g., Danshen), the anti-hyperlipidemia and anti-atherosclerotic effects of Zhilong Huoxue Tongyu Capsule (a patented TCM formula), the bioactive equivalent combinatorial components of the TCM Bupleuri Radix, and the molecular mechanism for melanin inhibitory activity of calycosin. In addition, a variety of studies related to microbial biodiversity, TCM clinical applications, development of animal models for TCM anti-anxiety research, as well as potential therapeutic benefits of natural products for treatment of neurodegenerative diseases were presented. These studies are all under the field of Pharmacology and Natural Product and Function.

Herein, we would like to express our great gratitude to have the honor as the organizer of Sino-CPLP Symposium on Natural Products and Biodiversity Resources (SNPBR) again. The 1st SNPBR was also held in Macao (SAR of China) in 2018, with the goal of advocating collaboration to promote novel perspectives and views in the field of biodiversity resources, medicinal plant research, and development of TCM research among basic scientists and industry partners from China and the Community of Portuguese Language Countries (CPLP) internationally. With the great support from scholars around the regions, the 2nd and 3rd SNPBR were held in Luzhou and Xi’an in China (Mainland) in 2019 and 2021, respectively. This time the 4th SNMBR had returned back to Macau, with over 100 keynote speeches and invited lectures by prestigious academics and scientists, as well as presentations and poster sessions prepared by young scientists and postgraduate students, all focusing on the frontier researches in pharmacology, medicinal chemistry, pharmacy, biodiversity resources, and industrial innovations in natural products as medicinal and functional foods, and TCM clinical application. Speakers had also drawn our attention to research innovations and laboratory management under the challenge of COVID-19 pandemic and beyond.

On behalf of the conference organizing committee, the editorial board would like to thank the Macau SAR government, the University of Macau, as well as the Macau Foundation for giving us the support. We would like to give our heartfelt gratitude and applause to all the speakers and participants who contributed to the success of the conference and their valuable works to this thematic collection. We would also like to thank the reviewers who offered their insightful help and constructive criticism for this publication. Finally, we thank the Editor-in-Chief of *Chinese Medicine*, Prof. Yitao Wang, for giving us the opportunity to prepare this special issue.

## A1 Microbial biodiversity of hypersaline environments as a target for bioprospection: Results from sampling in Cabo Verde and Portugal

### André Antunes^1,2,*^, Marta F. Simões^1^, Bryn McCulloch^3^, Cátia Santos-Pereira^4^, He Yang^1^, Sara C. Silvério^4^, Hélio Rocha^5^, Aires da Moura^5^, Lígia R. Rodrigues^4^

#### ^1^State Key Laboratory of Lunar and Planetary Sciences, Macau University of Science and Technology (MUST), Taipa, Macau SAR, China; ^2^Blue Marble Space Institute of Science, Seattle, USA; ^3^Edge Hill University, Ormskirk, UK; ^4^Centre of Biological Engineering (CEB), University of Minho, Braga, Portugal; ^5^Universidade Jean Piaget de Cabo Verde, Praia, Cabo Verde

##### **Correspondence:** André Antunes (aglantunes@must.edu.mo)

*Chinese Medicine *2023, **18(S2)**: A1

Hypersaline environments are abundant in arid, coastal and deep-sea locations. Despite their combination of multiple extremes, they host thriving microbial communities teeming with new species. Humankind has made use of microbes from such environments for several millennia (e.g., in the production of salt, sauces, and fermented foods). More recently, a rekindled interest in other applications led to an exponential rise in their use, which is now widespread across several industries.

In this work, we sampled different hypersaline sites in Portugal (Aveiro and Rio Maior) and Cabo Verde (islands of Boavista, Maio, and Sal). We included marine and non-marine salterns, and sites that are derelict and threatened due to the decline in economic relevance of salt production and urban pressure. Samples were collected, characterized, and used for cultivation-based studies and preliminary bioprospection. Initial results from Cabo Verde include the first data on physical–chemical characteristics (salinity, pH, temperature and ionic composition) for some locations, and relevant data on microbial metabolic profiling. Our data shows a wide diversity of niches (particularly in Maio) and uncovers differences in substrate use. Cultivation-based experiments resulted on isolation of Gammaproteobacteria, Firmicutes, and Archaea, with identification of likely novel taxa, while screening and genomic analysis pointed to potential biotechnological relevance of several isolates (e.g., production of enzymes, bioplastics, biominerals, or siderophores). Early results from Portuguese samples allowed the identification of strains exhibiting remarkable surfactant-like properties, with promising applications.

Our data confirms the high potential of these sites for isolating novel microorganisms with taxonomic and industrial significance. We expect further new findings from our continued analysis and future in-depth studies.

**Acknowledgements** Edge Hill University (RIF Project 1ANTUN16- EDEN), Fundação para a Ciência e a Tecnologia (FCT PTDC/BII-BIO/5554/2020), Science and Technology Development Fund of Macau (SKL-LPS(MUST)-2021–2023), Macau University of Science and Technology, and the State Key Laboratory of Lunar and Planetary Science (FRG grant No. FRG-22–079-LPS; FRG-22- RG-22–080-LPS; OPEN Project-ABAA).

## A2 Zebrafish as a drug screening platform for auditory sensory recovery and inner ear hair cell regeneration

### Andreia Ramos, Man Ieng Wong, Ieng Hou Lau, Flora Gordillo-Martinez, Raquel O. Vasconcelos*

#### Institute of Science and Environment, University of Saint Joseph, Macau, SAR, China

##### **Correspondence:** Raquel O. Vasconcelos (raquel.vasconcelos@usj.edu.mo)

*Chinese Medicine *2023, **18(S2)**: A2

The increasing levels of environmental noise due to growing urban aggregations are creating a serious hazard to the human auditory system, but also a wide range of adverse health effects including stress and anxiety disorders. Noise pollution is considered a threat to the public health especially in highly populated regions such as Macau, where there is also high prevalence of age-related diseases due to long-life expectancy. The Traditional Chinese Medicine (TCM) together with Chinese Herbal Medicine (CHM), have become an essential part of medical treatment (Zhang et al. 2022). TCM and CHM represent an extra dentary inventory of highly diverse compounds that promote health span and sensory-cognitive recovery. A few studies focusing on Noise-Induced Hearing Loss (NIHL) and Age-Related Hearing Loss (ARHL) have shown the potential of using TCM compounds as drug treatments for sensory recovery (Castañeda et al., 2019; Mahmoudian-Sani et al., 2019).

Our work has demonstrated that zebrafish can be used as a platform to investigate both NIHL and ARHL and the underlying structural–functional impairment and recovery at the inner ear level (Breitzler et al. 2020; Wong et al. 2022).

Noise treatment experiments with adult revealed noise level-dependent Temporal Threshold Shifts (TTS), auditory response latencies and recovery period. Hearing impairment was accompanied by hair cell loss and presynaptic activity decrease mostly above 150 dB re 1 μPa. Full regeneration of the sensory tissue occurred prior to functional recovery. The highest TTS were registered in continuous and fast intermittent noise treatments, and no differences were found between regular and irregular noise regimes. Aged individuals exposed to noise conditions showed the highest TTS.

Furthermore, transcriptomic analysis of the inner ear saccule (main auditory endorgan) of zebrafish adults with NIHL and ARHL pointed to differently expressed genes compared to young control group. Such genes were related to Extracellular matrix (ECM)-receptor pathways, which are known to play key roles in neuronal synaptic connections. Current research focuses on testing the effects of TCM compounds and underlying molecular pathways associated with sensory recovery from acquired hearing loss in the zebrafish model.

**References**

- Breitzler L, Lau I H, Fonseca P J, Vasconcelos R O. Noise-induced hearing loss in zebrafish: functional and structural inner ear damage and recovery. Hearing Research 2020; 391, 107952.
- Castañeda R, Natarajan S, Jeong S Y, Hong B N, Kang T H. Traditional oriental medicine for sensorineural hearing loss: Can ethnopharmacology contribute to potential drug discovery? Journal of Ethnopharmacology 2019; Vol. 231.
- Mahmoudian-Sani M R, Sheikhshabani S H, Mirfakhar F S, Asgharzade S. A review on medicinal plants used for treating ototoxicity and acoustic trauma induced hearing loss. Brazilian Journal of Pharmaceutical Sciences. 2019; 55.
- Wong M I, Lau I H, Gordillo-Martinez F, Vasconcelos R O. Impact of temporal variation in noise exposure on the auditory system and behavioural stress in the zebra8/7fish. Scientific Reports 2022; 12.
- Zhang Y, Xia Q, Wang J, Zhuang K, Jin H, Liu K. Progress in using zebrafish as a toxicological model for traditional Chinese medicine. Journal of Ethnopharmacology. 2022 282.

## A3 Orai3 regulation of Bk_ca_ channel trafficking through an Orai3-FOXO3-Unc13A signaling pathway in coronary artery vasomotion

### Hui Xiao^1,2^, Yang Fang^2^, Haoyang Lu^1^, Jizheng Guo^3^, Manyu Dai^1^, Yangcheng Xue^1^, Zhuoran Jia^1^, Lesha Zhang^2^, Bing Shen^2*^, Ren Zhao^1*^

#### ^1^Department of Cardiology, The First Affiliated Hospital of Anhui Medical University, Hefei, Anhui 230022, China; ^2^Department of Physiology, School of Basic Medical Sciences, Anhui Medical University, Hefei, Anhui 230032, China; ^3^Department of Pathophysiology, School of Basic Medical Sciences, Anhui Medical University, Hefei, Anhui 230032, China

##### **Correspondence:** Bing Shen (shenbing@ahmu.edu.cn); Ren Zhao (zhaoren@ahmu.edu.cn)

*Chinese Medicine *2023, **18(S2)**: A3

**Background:** Coronary artery spasm is a disease in which abnormal coronary artery smooth muscle cells (CASMCs) cause inappropriate vasoconstriction. Orai channel–mediated Ca^2+^ influx is important in vasoconstriction, whereas BK_Ca_ is a Ca^2+^-activated potassium channel involved in the negative regulation of vasoconstriction. A mechanistic understanding of the potential interactions between BK_Ca_ and Orai channels in coronary artery spasm is lacking.

**Methods:** Vessel tension measurement was used to verify the coronary vasoconstriction. The membrane potential of primary cultured CASMCs was identified by a fluorescent dye bis-(1,3-dibutylbarbituric acid) trimethine oxonol (DiBAC4(3)). The differential expressed proteins between Orai3 knockout (Orai3-KO) and wild-type rats were analyzed by liquid chromatography-mass spectrometry/mass spectrometry (LC–MS/MS). The cell membrane and cell cytoplasm components were separated and detected by western blot. The protein localization was detected by immunofluorescence experiment. Dual luciferase reporter assay was used to assess gene promoter.

**Results:** We show that BK_Ca_ channel function is decreased in coronary vasocontraction of Orai3-KO rats due to a problem in trafficking BK_Ca_ channels to CASMC membranes. We further show that BK_Ca_ channel trafficking requires Unc13A, but expression levels of this protein are downregulated in CASMCs derived from Orai3-KO rats. FOXO3 is a transcription factor for Unc13A. For FOXO3 to enter the nucleus, it must first be dephosphorylated, a process that we show is dependent on Orai3-mediated Ca^2+^ signaling through calmodulin and calcineurin.

**Conclusion:** We propose a novel Orai3-FOXO3-Unc13A signaling pathway that participates in the regulation of vasomotion and provides potential therapeutic targets for coronary vasoconstriction disorders.

**Acknowledgments** The authors thank the Comprehensive Experiment Center of Basic Medical Sciences and the Center for Scientific Research of Anhui Medical University for valuable support of this work. This study was supported by the Natural Science Foundation of China (Grant Nos. 81970446, 81570403, U22A20272).

## A4 TCM, from molecular to clinical application: challenges and constraints

### Diogo Calado^1*^, Antonio Moreira^2,3^, Silvia Pocas^4^, Rui Goncalves^4^

#### ^1^International Education College, Tianjin University of Traditional Chinese Medicine, Tianjin, China; ^2^Portuguese Society of Chinese Medicine, Rio Maior, Portugal; ^3^Sports Sciences School of Rio Maior, Polytechnic Institute of Santarem, Santarem, Portugal; ^4^Portuguese Red Cross North Health Sciences School, Oliveira do Hospital, Portugal

##### **Correspondence:**: Diogo Calado (info.tcm@gmail.com)

*Chinese Medicine *2023, **18(S2)**: A4

**Main text**

Since 1996, Traditional Chinese Medicine (TCM) in China has undergone a continuous process of modernisation and innovation. The identification of compounds, their structures and properties, as well as the analysis and study of the relationship between different compounds and substances has allowed a better understanding of the mechanisms of action of Chinese medicine [medication/herbal therapy] and the optimisation of therapeutic effectiveness and the development of its formulas, improving its integration with Western medicine, while increasing the level of available evidence.

On the other hand, as more evidence accumulates, after being evaluated by the European Medicines Agency, more substances and formulas are moved from dietary supplements category to medicines category.

According to recent Portuguese law, the education of TCM is now at higher education, which should provide technical and deontological autonomy, enabling decision-making capacity and professional independence. In this exploratory study, the education content, number of academic credits and legal framework of various healthcare courses at Portuguese higher education, as well as which of them permit dietary supplements or medicines prescription, will be analysed. The academic background of the teaching staff and internship tutors of TCM-related bachelor’s degree courses will also be studied.

The results raise doubts about the prospect of current and future TCM practitioners having the required academic knowledge to prescribe Chinese medicine, when and if they are included in medicines category. Additionally, a question arises whether Portuguese higher education institutions and its teaching staff currently have the know-how and academic background to teach this scientific area at a level adequate for medicine prescription.

In conclusion, ways to overcome these constraints shall be discussed, in order to allow future TCM professionals to acquire the necessary skills to prescribe Chinese medicine in medicines category.

**Keywords**: Chinese medicine; modernisation; higher education; clinical training; prescription.

## A5 Beneficial effects of novel tetramethylpyrazine derivative (T-006) on cognitive impairment in AD-related mouse models

### Guiliang Zhang^1,2^, Zaijun Zhang^2^, Maggie Pui Man Hoi^1*^

#### ^1^Institute of Chinese Medical Sciences, State Key Laboratory of Quality Research in Chinese Medicine, University of Macau, N22-7012, Avenida da Universidade, Taipa, Macau, SAR, China; DPS, Faculty of Health Sciences, University of Macau, Macau, SAR, China; ^2^Institute of New Drug Research, International Cooperative Laboratory of Traditional Chinese Medicine Modernization and Innovative Drug Development of Chinese Ministry of Education, Jinan University College of Pharmacy, Guangzhou, China

##### **Correspondence:** Maggie Pui Man Hoi-MagHoi@um.edu.mo

*Chinese Medicine *2023, **18(S2)**: A5

**Background:** Alzheimer’s disease (AD) is an age-related progressive neurodegenerative disease currently lacks effective treatment. T-006, a small molecule compound derived from tetramethylpyrazine (TMP), has shown potential as therapeutic drug for neurodegenerative diseases in our previous studies.

**Materials and methods:** To investigate the prophylactic treatment effect of T-006 for AD, T-006 (3 mg/kg) was applied as preventive treatment to two AD-related transgenic mouse models, 2xTg (APP/PS1) (2-month-old; *n* = 8–10) and 3xTg (APP/PS1/MAPT) (4-month-old; *n* = 8–10) for 6 and 8 months, respectively. Behavioral tests including Step-down avoidance (SDA), Morris Water Maze (MWM) and Novel Object Recognition (NOR) were used to evaluate the cognitive functions of the mice. Amyloid-beta (Aβ) and hyperphosphorylated tau (p-Tau) depositions and related proteins in the underlying processing pathways were evaluated by western blot (WB). The drug target of T-006 was identified by using Drug Affinity Responsive Target Stability (DARTS).

**Results:** Our studies showed that T-006 improved behavioral performance of AD-related mouse models, enhancing their cognitive abilities for learning, including spatial memory (MWM), cognition memory (NOR), and emotional memory (SDA). Western blot evaluation of the brain tissues showed that T-006 attenuated Aβ deposition through inhibiting amyloid-beta precursor protein (APP) production and beta-secretase 1 (BACE-1) activity. In 3xTg mice T-006 also effectively reduced the production of p-Tau and total Tau and improved the regulation of autophagy while increasing the expression of synapse-associated proteins. In addition, T-006 modulated the JNK and mTOR-ULK1 pathways to reduce both p-tau and total tau levels. DARTS analysis showed that T-006 targeted mitochondrial-related protein alpha-F1-ATP synthase (ATP5A).

**Conclusion:** Our data suggested that T-006 mitigated cognitive decline by reducing Aβ deposition through reducing APP production, reducing p-tau and total tau through autophagy-related JNK and mTOR-ULK1 signaling pathways, supporting further investigation into its development as a candidate drug for AD treatment.

**Acknowledgements ** The authors thank the Science and Technology Development Fund (Macau SAR) (0015/2019/ASC, 0023/2020/AFJ, 0035/2020/AGJ) and the University of Macau Research Grant (MYRG2022-00248-ICMS, MYRG-CRG2022-00010-ICMS).

## A6 Theoretical exploring of compounds with anti-thrombosis effect from *Salvia miltiorrhiza* in zebrafish

### Jiahui Zhu^1,†^, Huilan Tang^2,†^, Ningyi Qin^3^, Chang Rao^1^, Haiqiang Wang^1^ and Guang Hu^1*^

#### ^1^School of Pharmacy and Bioengineering, Chongqing University of Technology, Chongqing 400054, China; ^2^Chongqing Institute for Food and Drug Control, Chongqing 401121, China; ^3^Chongqing Pharmaceutical Group Huamosheng Pharmaceutical Science & Technology Co., Ltd., Chongqing 400050, China

*Chinese Medicine *2023, **18(S2)**: A6

##### **Correspondence:** Guang Hu Hu-foxhu@cqut.edu.cn

† Two authors contributed equally to the paper.

Computer virtual screening techniques such as molecular docking are helpful tools in drug discovery progress, due to their high throughput screening characteristics which make them suitable to deal with a large number of molecular structures binding to the drug targets. Zebrafish, an effective in vivo model for discovering potential active molecules, can support with feasible data due to the high genetic, physiologic, and pharmacologic similarity with humans. Combined molecular docking and zebrafish screening are applied in predicting and confirming the anti-thrombosis compounds in Danshen (DS), which is the dry root of *Salvia miltiorrhiza Bge.* and has been used in traditional Chinese medicine (TCM) for many years.

Over 202 compounds in DS were docked with 24 thrombosis-relevant target proteins, to predict the potential active ingredients by calculating the scores of docking between DS ingredients and thrombosis-related proteins. Afterward, commercially obtained chemical standards of the compounds with high scores were tested in a chemical-induced zebrafish thrombosis model.

Four compounds in DS including salvianolic acid B, lithospermic acid, rosmarinic acid, and luteolin-7-O-β-D-glucoside which were calculated with high scores by molecular docking exhibited a dose-dependent anti-thrombotic effect in Adrenaline hydrochloride (AH)/phenylhydrazine (PHZ)-induced zebrafish thrombosis model by both pre-treatment and post-treatment methods.

Our research demonstrated a feasible way of exploring potential active compounds from TCM by combining molecular docking and zebrafish model screening methods. Moreover, our study could provide references for future investigation of the anti-thrombotic effects of DS.

## A7 Effects of anxiolytics diazepam and buspirone on anxiety-like behaviors induced by Liang’s contextual-stress box in mice

### Tian-Ge Zheng, Jing-Yi Jia, Zhe-Yu Hu, Zhi-Bo Zhang, Zhi-Hui Cheng, Zhong-Rui Wang, Xiao-Long Shen, Jian-Hui Liang^*^

#### Department of Molecular and Cellular Pharmacology, Peking University School of Pharmaceutical Sciences, Beijing 100083, China

##### **Correspondence:** Jian-Hui (Liang-liangjh@bjmu.edu.cn)

*Chinese Medicine *2023, **18(S2)**: A7

**Background:** Liang’s contextual-stress box (Liang’s Box) is designed and invented based on the ‘approach-avoidance conflict theory’ to induce anxiety-like behaviors, especially Liang’s Restless Posture (LRP) that is defined as the flat-back body stretching and hindleg abducting with or without creeping movement, head-dipping or sniffing in mice ^[1, 2]^. This study investigated the effects of diazepam and buspirone on characteristic behaviors induced by Liang’s Box primarily to assess its predictive validity.

**Methods:** Liang’s Box consisted of a central area and the peripheral area of three arms (Fig. 1). Spatiotemporal data and ethological indexes collected in Liang’s box were measured and compared with the counterparts induced by the classical experimental paradigms the Elevated Plus-Maze (EPM) and the Open Field (OF) test. Diazepam (0.5, 1.0, and 2.0 mg·kg^−1^) and buspirone (0.5, 2.0, and 10 mg·kg^−1^) were injected to mice intraperitoneally and the following behavioral indexes were recorded for 15 min in Liang’s Box.

**Figure 1 (abstract A7):**
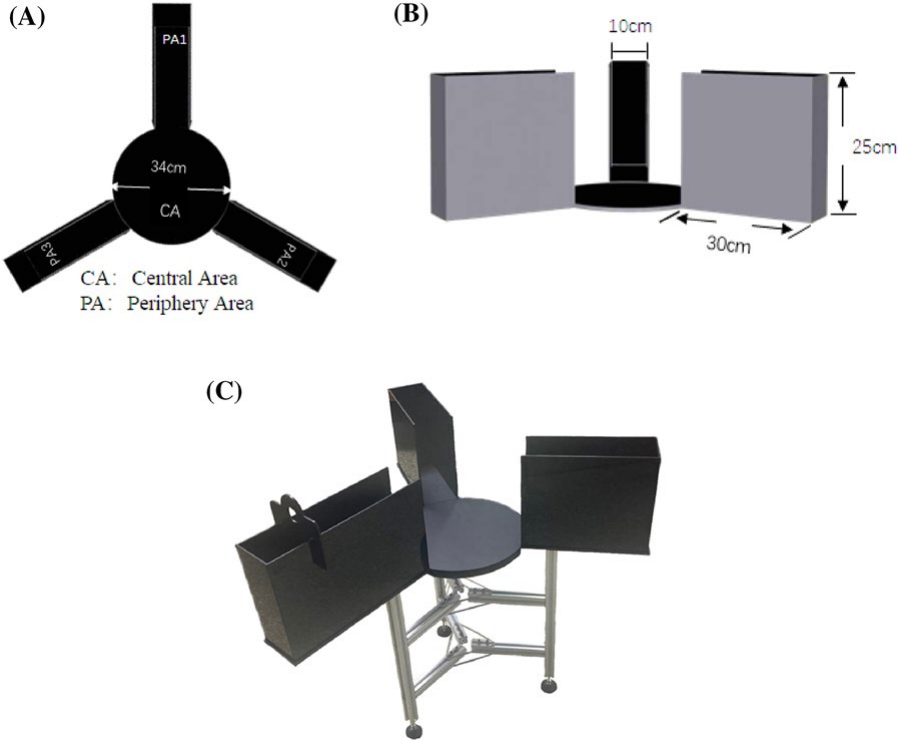
Design and structure of the Liang’s contextual stress box. A: Schematic diagram; B: Side view; C: Bird′s-eye view.

**Results:** 1. The duration and frequency of LRP induced by Liang’s box were significantly longer and higher than those of EPM and OF. Compared with EPM and OF, Liang’s box was of the simple experimental procedure, the moderate structural conflicts and the widened exploration areas of the model device, the definitive division of sub-regions, and less statistical dispersions; 2. 0.5, 1.0 and 2.0 mg·kg^−1^ of diazepam and 0.5, 2.0 and 10 mg·kg^−1^ of buspirone could significantly and dose-dependently reduce the duration and frequency of LRP in mice; 3. 0.5 and 2.0 mg·kg^−1^ of diazepam statistically increased the travel distance and time in the central area, transition in zones, global activity, frequency and duration of head-dipping, and total travel distance in Liang’s box.

**Conclusions:** The anxiety-like behavioral syndrome can be induced by Liang′s box in mice, which may be used as a novel experimental paradigm of anxiety to explore its pathophysiological mechanisms, as well as screening and assessing certain new anxiolytics.

**Acknowledgements** This study was supported by the National Natural Science Foundation of China (Grant Numbers: 82173799; to Jian-Hui Liang).

**References**

1. LIANG Hui, GONG Qi, CHENG Tao, LIANG Jian-hui. Effect of buspirone and mianserine on anxiety- and depression-like behaviors induced by Liang′s contextual-stress box in mice. Chin J Pharmacol Toxicol. 2017; 31(2): 131–137.
2. CHENG Tao, ZHANG Qing-jie, ZHENG Tian-ge, LI Yun-feng, LIANG Jian-hui. Depression-like behaviors induced by Liang′s contextual-stress box in mice. Chin J Pharmacol Toxicol. 2020; 34(2): 125-132.

## A8 Treatment of Biomarker-Enriched Advanced Hepatocellular Carcinoma with Icaritin as a Small Molecule Immune Modulator

### Shukui Qin, Hong Guo, Daying Yang, Gang Chen, Junma Zhou, Bin Ye, Kun Meng*, and Yan Sun

#### Bejing Shenogen Pharma Group, Beijing, China

##### **Correspondence:** Kun Meng (kunm88@shenogen.com)

*Chinese Medicine* 2023, **18(S2)**: A8

Icaritin is a prenylflavonoid derivative obtained from Epimedium genus that has long been used in Chinese traditional medicine. It has been demonstrated to exert pleiotropic pharmacological and biological activities including anti-inflammation and tumor inhibition. Based on its therapeutic effects in cancer inhibition, Icaritin had entered clinical trials for the treatment of advanced hepatocellular carcinoma (HCC). In phase III adaptive trial, randomized, double-blinded, multicenter study of Icaritin versus Huachansu was performed since September 2017, in which a composite biomarker score (CBS) consisting of AFP, TNF and IFNγ was used for patient stratification.

In total, 543 participants were screened, and 283 patients were randomly assigned (1:1) to the Icaritin and Huachansu treatment groups. The interim analysis of 71 CBS-positive patients demonstrated a median overall survival of 13.54 months (95% CI, 7.36–20.07, n=33) versus 6.87 months (95% CI, 4.93–11.43, n=38) Icaritin and Huachansu groups (HR = 0.43, 95% CI 0.23–0.82, p = 0.0092). Grade ≥ 3 treatment-related adverse events (TRAEs) including elevation in serum γ-glutamyltransferase (2.8%) were observed in 12.1% of Icaritin-treated patients (n=141), and TRAEs including diarrhea (7.1%) were observed in 26.2% of Huachansu-treated patients (n=141). QOL were 7.3 months vs. 2.7 months observed in patients with Icaritin and control treatments.

We previously reported that Icaritin inhibited IL-6 signaling pathway. To further elucidate the underlying mechanisms of tumor inhibition, multiple approaches had been applied in more studies. We obtained evidence that Icaritin directly interacts with MyD88 and IKK proteins, indicating Icaritin interferes with both MyD88 pathway and NF-κB pathway. Since MyD88 and NF-κB are both involved in innate and adaptive immune responses, we examined Icarritin’s impact on tumor immune environment. We proposed that immune modulation mechanisms underlie Icaritin’s tumor inhibition effect.

## A9 Study of Antioxidant Capacity of Solutions from *Lycium Ruthenicum* Murr. Soaked at Various Conditions

### Veng Meng Lo^1*^, Tik ManWong^1^, Chi On Tam^1^

#### ^1^Faculty of Health Sciences and Sports, Macao Polytechnic University, Macao, SAR, China

##### **Correspondence:** Veng Meng Lo (vmlo@mpu.edu.mo)

*Chinese Medicine* 2023, **18(S2)**: A9

**Background:** *Lycium ruthenicum* Murr., which belongs to the classification of Lyceum of Solanaceae, has high content of anthocyanin. Anthocyanin is well-known of its water-soluble free radical scavenger effect. A vast number of studies have mentioned antioxidant components and their antioxidant abilities[1,2].

**Purposes:** Elevated temperature or prolonged soaking may destroy effective antioxidant ingredients. This study analyzed the antioxidant release and antioxidant ability under different soaking conditions (temperature and time).

**Methods:** Soak 5g of fruit in 200ml of distilled water at the temperature of 4°C, 25°C and 55°C and the time is 0, 2, 4, 6 and 12 hours respectively. The antioxidant ability was evaluated with: ABTS(2, 2-azinobis-(3-ethylbenzthiazoline-6-sulfonic acid) diammonium salt, FRAP(Ferric Reduction Ability Plasma assay), OH(Hydroxyl Radical Scavenging assay) and DPPH(2,2-diphenyl-1-picryl-hydrazyl-hydrate free radical scavenging rate tests). Analysis of main antioxidant components by HPLC(High Performance Liquid Chromatography) method.

**Results: **

- The main antioxidant in *Lycium ruthenicum* Murr. is petunidin. It accounts 88% of the total anthocyanins.
- [4] The longer the soaking time, the more antioxidant components are released. But it is not just a simple proportional relationship (Table 1). After 2-hour of soaking, the releasing rate slows down. The antioxidant ability is also increased with the soaking time within 2-hour (Table 2).
- [5] Soaking in high temperature for 2-hour, the antioxidant ability (except DPPH) is stronger, but after at, various antioxidant ability tests showed different changes (Table 2). The content of petunidin soaked in high temperature is the lowest, and the peak value appears at 2- hour. However, other anthocyanins (e.g., malvidin) are obtained differently (Table 1).

**Conclusion:** The anti-oxidative ability of the soaking solution increases with time and temperature. But this is not the simple proportional relationship. Due to the different release and decomposition modes of various antioxidant agents under different immersion conditions, the antioxidant ability undergoes complex variation.

**Table 1 (abstract A9):** Anthocyanin content after different soaking conditions

Anthocyanin	Petunidin (mg/mL)			Malvidin (mg/mL)		
Time\Temp	4℃	25℃	55℃	4℃	25℃	55℃
0 h	0.002	0.000	0.016	0.000	0.000	0.001
2 h	0.124	0.122	0.076	0.006	0.007	0.016
4 h	0.191	0.228	0.057	0.008	0.010	0.017
6 h	0.222	0.298	0.046	0.011	0.013	0.019
12 h	0.313	0.400	0.030	0.018	0.020	0.020

**Table 2 (Abstract A9):** Antioxidant ability after different soaking conditions

Test	ABTS (mM)			FRAP (mM)			OH (%)			DPPH (%)		
Time\Temp	4℃	25℃	55℃	4℃	25℃	55℃	4℃	25℃	55℃	4℃	25℃	55℃
0 h	0.315	0.319	0.346	1.387	1.226	1.3	17.1	15.5	36.0	121.8	88.3	63
2 h	0.347	0.349	0.438	3.812	3.900	5.6	62.4	60.5	66.2	103.2	89.8	15
4 h	0.350	0.353	0.334	4.469	4.751	5.2	64.3	52.9	58.2	96.6	75.7	42
6 h	0.352	0.358	0.281	5.199	5.434	5.2	53.7	37.8	54.5	85.9	82.9	71
12 h	0.358	0.359	0.248	6.573	6.229	4.8	48.6	36.5	49.7	78.4	73.9	43

**References**

1. Guo J,Tao L,Wang R,Wang J,Ren J, Ren H Research Progress on Nutritional Components and Development of Lycium ruthenicum Murr. Food Sci. Technol. 2022; 2: 112–114
2. Cui SZ, Xie XL, Jia DS, Li RQ, Wen CX, Liu LD, H JX Antioxidant Activity of Extracts from Different Solvents of Black Wolfberry Food Res. And Dev. 2017; 38(9): 38–41

## A10 Amyloid-beta Skews Astrocytes from Being Supportive to Detrimental and the Complicated VEGF Signaling in the Blood-Brain Barrier

### Qian Yue, Maggie Pui Man Hoi^*^

#### Institute of Chinese Medical Sciences, State Key Laboratory of Quality Research in Chinese Medicine, University of Macau, N22-7012, Avenida da Universidade, Taipa, Macau, SAR, China; DPS, Faculty of Health Sciences, University of Macau, Macau, SAR, China

##### **Correspondence:** Maggie Pui Man Hoi (maghoi@um.edu.mo)

*Chinese Medicine *2023, **18(S2)**: A10

Astrocytes are very capable secretors of cytokines and chemokines along with other trophic and morphogenic factors essential for their microenvironments. Astrocytes are in constant crosstalks with other cells such as microglia, perivascular cells, and blood-borne leukocytes. Depending on the timing and context, reactive astrocyte stimulated by brain insults may exacerbate inflammatory reactions or promote immunosuppression. As an integral component of the BBB, perivascular astrocytic endfeet enwrap blood vessels that are composed of brain endothelial cells (BECs) surrounded by pericytes and basement membrane. In AD patients, pericyte number and coverage are greatly reduced (60% and 30%) with disrupted PDGFβ/PDGFRβ signaling. Concomitantly, astrocytes become reactive, endfeet become swollen and detached from vessels, phagocytic ability for Aβ is increased but neurotransmitter reuptake is decreased, and astrocytic secretions skewed to proinflammatory profiles with high levels of cytokines, chemokines, nitric oxide (NO), ROS, vasoactive agents and MMPs. Our recent study showed endothelial disintegration was the effect of secretions from reactive astrocytes but not direct Aβ action. Aβ-treated astrocyte medium downregulated tight junctions (TJ) and adherens junctions (AJ) and increased vascular permeability. Efflux transporters (P-gp and BCRP) were also decreased, but unexpectedly endothelial VEGFR2 signaling was suppressed. Astrocytic VEGFA has been reported to cause BBB hyperpermeability in various CNS inflammatory diseases. Interestingly, beneficial effects of VEGF has also been reported for stabilization of neovascularization. We also observed suppressed VEGFR2 (pY1175) signaling in BECs treated with conditioned medium of Aβ-stimulated astrocytes but not by Aβ. This prompts us to speculate that Aβ skews astrocytes to an imbalance of secretary factors that obstructs BECs viability. The relationship between astrocytic secretome and BBB functions in AD warrants further investigation.

**Acknowledgements** This work was supported by grants from FDCT (0015/2019/ASC, 0023/2020/AFJ, 0035/2020/AGJ) and UMRG (MYRG2015-0061-ICMS-QRCM, MYRG2017-00150-ICMS).

## A11 Zhilong Huoxue Tongyu Capsule Inhibits Hyperlipidemia and Atherosclerosis Through NF-ΚB/NLRP3 Signaling Pathway

### Mengnan Liu^1,2^, Gang Luo^1^, Mingtai Chen^2,3^, Zhongjing Hu^2,3^, Mei Han^2,3^, Maryam Mazhar^1^, Qibiao Wu^2^, Sijin Yang^1,3*^

#### ^1^National Traditional Chinese Medicine Clinical Research Base and Department of Cardiovascular Medicine, the Affiliated Traditional Chinese Medicine Hospital of Southwest Medical University, Luzhou, Sichuan, P.R. China; ^2^Faculty of Chinese Medicine and State Key Laboratory of Quality Research in Chinese Medicine, Macau University of Science and Technology, Macau SAR, P.R. China; ^3^Department of Cardiovascular Disease, Shenzhen Traditional Chinese Medicine Hospital, Shenzhen, Guangdong, P.R. China

##### **Correspondence:** Sijin Yang (ysjimn@sina.com)

*Chinese Medicine *2023, **18(S2)**: A11

Corresponding author at the Affiliated Traditional Chinese Medicine Hospital of Southwest Medical University.

**Background:** Hyperlipidemia and atherosclerosis (AS) are gradually developing but devastating diseases with increasing incidence worldwide. In China, the treatment methods based on integrated traditional Chinese and western medicine often provide better curative effect on diseases. Zhilong Huoxue Tongyu Capsule (ZL) is a Chinese patent medicine used to treat cardio-cerebral diseases. However, the pharmacological mechanism by which it regulates blood lipids and treats AS remains unclear. The purpose of this study is to explore the mechanism of ZL inhibiting hyperlipidemia and AS.

**Methods:** Fifty New Zealand white rabbits were equally divided into the control, model, model + ZL (3.12 g/kg/d, i.g.), model + atorvastatin (0.51 mg/kg/d, i.g.) and model + ZL + atorvastatin groups. Except for the control group, all other groups underwent carotid intima air drying and received a high-fat diet for 28 days to establish hyperlipidemia AS model, and drug treatment was given for the same period of time after modeling. Morphological changes and blood lipids were detected, NF-κB/NLRP3-related protein or gene expression levels were analyzed in carotid tissue.

**Results:** ZL significantly reduced blood lipids and delayed the progression of AS. TC, TG and LDL-C were decreased while HDL-C was increased in blood, IMT thickening and plaque formation of carotid arteries were inhibited, VRI was alleviated, and morphological features were improved. NF-κB, NLRP3 and IL-1β in the carotid artery were significantly down-regulated after intervention with ZL. RT-PCR and western blot analysis showed that NF-κB (p-NF-κB), NLRP3, Caspase-1, IL-1β and IL-18 were significantly downregulated by ZL.

**Conclusion:** ZL can be used effectively as adjuvant therapy for hyperlipidemia and AS, combining it with atorvastatin yielded more optimized efficacy, but its anti-inflammatory and pharmacological mechanisms of inhibiting pyroptosis should be studied further.

**Keywords:** Zhilong Huoxue Tongyu capsule, hyperlipidemia, atherosclerosis, NF-κB/NLRP3 signaling pathway, traditional Chinese medicine

**Figure 1 (abstract A11):**
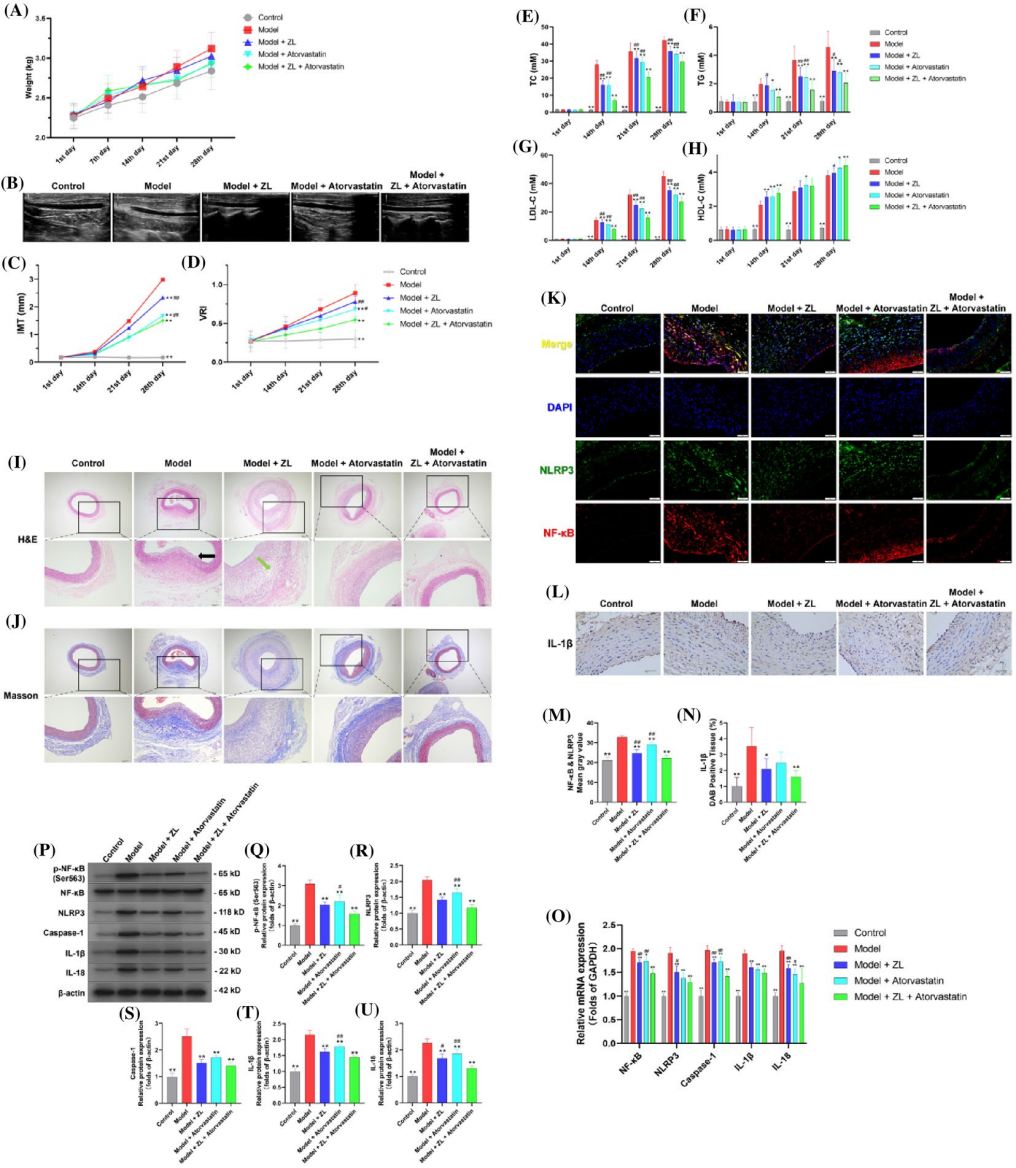
ZL improve the AS in rabbits. (A) The weight changes of all experimental animals within 28 days, and the analysis found that there was no statistical significance in the comparison of weight among the groups, the line chart showed the trend of weight growth, bars represent the mean ± SD, n = 10. (B) Original image of carotid artery detected by color Doppler ultrasound. Doppler ultrasound demonstrated the changes of (C) IMT and (D) VRI, and the measurements data on day 28th were used for statistical analysis. The automatic biochemical analyzer is used to detect the blood lipid content in the collected blood samples, (E) TC, (F) TG, (G) LDL-C and (H) HDL-C. Bars represent the mean ± SD, n = 10. (I) H&E staining, (J) Masson staining. Under the microscope, 40 × images were acquired to show histological changes across the entire vessel cross-section, with 100 × magnification to reveal details. (K) Double immunofluorescence of NF-κB and NLRP3. Atherosclerotic tissue was stained with NF-κB (green), NLRP3 (red) and DAPI (blue) to mark atherosclerotic plaques and areas of inflammation, respective change in color in merged figures corresponds to Red + Blue = Magenta; Red + Green = Yellow. (L) Immunohistochemistry of IL-1β. For the above staining, we made two slices, and three fields of view (× 200) were randomly selected for each slice for gray value analysis. (M) Gray value analysis of Double immunofluorescence (NF-κB & NLRP3). (N) Gray value analysis of Immunohistochemistry (IL-1β). Bars represent the mean ± SD, n = 6. (O) Graphs showed relative expression levels of mRNA, including NF-κB, NLRP3, Caspase-1, IL-1β, and IL-18. Bars represent the mean ± SD, n = 6. Western blotting demonstrated ZL downregulated the protein expression of NF-κB/NLRP3 signaling in atherosclerotic tissue. (P) A representative immunoblot, (Q) p-NF-κB, (R) NLRP3, (S) Caspase-1, (T) IL-1β, (U) IL-18. Bars represent the mean ± SD, n = 3. *p < 0.05 and **p < 0.01 as compared to model group, #p < 0.05 and ##p < 0.01 as compared to model + ZL + atorvastatin group

## A12 Bioactive Equivalent Combinatorial Components for quality consistency assessment for multi-originated Chinese herbal medicine Bupleuri Radix

### Mingming Zhao^1^, Linxuan Xiao^1^, Qiling Chen^1^, Liyu Shen^1^, Guanding Zhao^1^, Ke-Gang Linghu^1^, Jinyu Yu^1^, Parsa Dar^1^, Qiushuo Ma^1^, Hua Yu^1,2*^

#### ^1^Institute of Chinese Medical Sciences, State Key Laboratory of Quality Research in Chinese Medicine, University of Macau, Macao, China; ^2^Macao Centre for Research and Development in Chinese Medicine, Institute of Chinese Medical Sciences, University of Macau, Macao SAR, China

##### **Correspondence:** Hua Yu (bcalecyu@um.edu.mo)

*Chinese Medicine *2023, **18(S2)**: A12

Bupleuri Radix (BR) is one of the widely used herbal medicine in China for fever and inflammation associated with influenza. *Bupleuri chinense* DC (BCH) and *B. scorzonerifolium* Willd (NCH) are two plant species recommended by the current Chinese Pharmacopoeia for BR. However, the quality control standard in Chinese Pharmacopoeia is well described for BCH but not clear for NCH. Therefore, the current study aims to perform a comparative investigation using the Bioactive Equivalent Combinatorial Components (BECCs) approach for BCH and NCH, thus providing reasonable chemical markers for further improvement of the quality control method for BR.

Based on the study, the combinations of 18 and 21 compounds were discovered as BECCs for BCH and NCH, respectively. The in vitro (anti-inflammatory effects in LPS-induced RAW264.7 macrophages) and in vivo (antipyretic effects in LPS-induced fever in rat) studies indicated the developed chemical combinations could be representatives for the BCH and NCH extracts, respectively. Moreover, 11 compounds from the BECCSs were investigated to contribute to the anti-inflammatory and antipyretic activities of BR.

This work provides a systemic investigation and comparative comprehension on the chemical and biological features between BCH and NCH, and provides the potential chemical markers for the quality control of BR and related products.

**Acknowledgments**

This study was financially supported by the Science and Technology Development Fund, Macau SAR [FDCT No. 0096/2019/A2, No. 0058/2020/AGJ, No. 0159/2020/A3,], and the Research Committee of the University of Macau [2022-00189-ICMS].

**Ethics Approval**

The animal study protocol was approved by the Institutional Review Board (or Ethics Committee) of University of Macao (NO. UMARE-004-2022, approved on 4 March 2022).

## A13 Molecular docking, molecular dynamics simulation, and free energy computations in investigation of the melanin inhibitory activity of calycosin in zebrafish

### Nilupaier Tayier^1,†^, Ningyi Qin^2,†^, Yi Zeng^1^, Yu Wang^1^, Guang Hu^1*^

#### ^1^School of Pharmacy and Bioengineering, Chongqing University of Technology, Chongqing 400054, China; ^2^Chongqing Pharmaceutical Group Huamosheng Pharmaceutical Science & Technology Co., Ltd., Chongqing 400050, China

^†^The two authors contributed equally to the paper.

##### **Correspondence: **Guang Hu (foxhu@cqut.edu.cn)

*Chinese Medicine 2023*, **18(S2)**: A13

Calycosin, an active isoflavone component in Radix Astragali, was proved to promote angiogenesis in zebrafish (*Danio rerio*) larvae in our previous study. Occasionally, it is found that the whole blood vessel restore progress could be observed directly without treatment of 1-phenyl 2-thiourea (PTU), a tyrosinase inhibitor usually applied to block pigmentation and keep the transparency in zebrafish embryo. This phenomenon might suggest that calycosin itself could suppress melanogenesis via certain mechanisms.

By comparing with the activities of natural products known for anti-melanogenesis effects, such as kojic acid and arbutin, the inhibitory effect of calycosin on melanin production was confirmed in a zebrafish in vivo model. Furthermore, the kinetics and the mechanisms of calycosin binding to tyrosinase were performed using computer virtual techniques, such as molecular docking, molecular dynamic simulations, and free energy analysis.

The results of in vivo study showed that calycosin could significantly inhibit zebrafish (48 hpf) pigmentation at the concentration ranging from 7.5 to 30 µM, with a lower IC_50_ of 30.35 µM compared to arbutin, kojic acid and hydroquinone. Moreover, data acquired from molecular docking, molecular dynamics simulations, and free energy analysis indicated that calycosin could bind to tyrosinase with high binding affinity.

Our study suggested that calycosin could inhibit zebrafish pigmentation during the drug treatment period, and the mechanism of action might be related with its good binding affinity to tyrosinase.

## A14 Role of GJA1 in proliferation and contraction of basilar artery smooth muscle cell in high salt environment

### Wen Ding^1#^, Pingping Wang^1#^, Qinggang Meng^2^, Shaobo Ma^1^, Fan Zheng^1^, Xinyang Hu^1^, Wenyang Chen^3^, Qitian Mu^4^, Hongbo Chen^2*^, Bing Shen^1*^

#### ^1^School of Basic Medical Sciences, Anhui Medical University, Hefei, Anhui 230032, China; ^2^Department of Obstetrics and Gynecology, Maternal and Child Health Hospital, the Affiliated Hospital of Anhui Medical University, Hefei, Anhui 230001, China; ^3^Central Laboratory, Fujian Medical University Union Hospital, Fuzhou, Fujian 350001, China; ^4^Laboratory of Stem Cell Transplantation, Ningbo First Hospital, Ningbo, Zhejiang 315010, China

^#^ These authors contributed equally.

##### **Correspondence:** Hongbo Chen (chenhongbo@ahmu.edu.cn); Bing Shen (shenbing@ahmu.edu.cn)

*Chinese Medicine *2023, **18(S2)**: A14

**Background:** Cerebrovascular spasm is a life-threatening event in salt-sensitive vascular disease. The effect of high-salt environment on vasoconstriction disorder has not been fully clarified.

**Methods:** Immunoblotting, proliferation assay and vessel tension measurement were used.

**Results:** Additional NaCl adding into culture medium enhanced the expression ratio of phosphorylated gap junction protein alpha 1 (GJA1)/GJA1 and proliferation in rat cerebral basilar artery smooth muscle cells (CBASMCs), but there were not caused by high-salt-induced osmotic pressure increase. Knockdown of GJA1 by transfection with GJA1-specific siRNA or inhibition of GJA1 S368 phosphorylation significantly inhibited CBASMCs proliferation. High-salt medium culture with additional 40 mM NaCl enhanced GJA1 S368 phosphorylation and U46619- and endothelin 1-induced contraction of cerebral basilar artery. Knockdown of GJA1 by transfection with GJA1-specific siRNA inhibited agonist-induced contraction, but inhibition of GJA1 S368 phosphorylation did not in rat cerebral basilar artery which was cultured in normal-salt or high-salt medium.

**Conclusion:** We demonstrated that high-salt environment-induced GJA1 phosphorylation enhanced CBASMCs proliferation, but did not affect cerebral basilar artery vasoconstriction. GJA1 involved in CBASMCs proliferation and cerebral basilar artery vasoconstriction, and may be a potential therapeutic target for cerebrovascular disorder induced by high-salt environment.

**Acknowledgments** We would like to express our gratitude to Yunxia Lu, Ph.D. (and the technicians), in the Comprehensive Experiment Center of Basic Medical Sciences, Anhui Medical University, for the support of these facilities, and the Center for Scientific Research of Anhui Medical University for valuable assistance with this study.

